# Galerkin finite element analysis of magneto two-phase nanofluid flowing in double wavy enclosure comprehending an adiabatic rotating cylinder

**DOI:** 10.1038/s41598-021-95846-2

**Published:** 2021-08-13

**Authors:** Wael Al-Kouz, Bilal Abdel-Illah Bendrer, Abderrahmane Aissa, Ahmad Almuhtady, Wasim Jamshed, Kottakkaran Sooppy Nisar, Abed Mourad, Nawal A. Alshehri, Mohammed Zakarya

**Affiliations:** 1 Department of Mechanical and Maintenance Engineering, German Jordanian University, Amman, 11180, Jordan; 2 Laboratoire de Physique Quantique de la Matière et Modélisation Mathématique (LPQ3M), University of Mascara, Mascara, Algeria; 3 Department of Mathematics, Capital University of Science and Technology (CUST), Islamabad, 44000 Pakistan; 4 Department of Mathematics, College of Arts and Sciences, Prince Sattam bin Abdulaziz University, Wadi Aldawaser, 11991 Saudi Arabia; 5 Department of Mathematics and Statistics, College of Science, Taif University, P.O. Box 11099, Taif, 21944 Saudi Arabia; 6 Department of Mathematics, College of Science, King Khalid University, P.O. Box 9004, Abha, 61413 Saudi Arabia; 7 Department of Mathematics, Faculty of Science, Al-Azhar University, Assiut, 71524 Egypt

**Keywords:** Mathematics and computing, Physics

## Abstract

In this work, the finite element method is employed to simulate heat transfer and irreversibilities in a mixed convection two-phase flow through a wavy enclosure filled with water–alumina nanoliquid and contains a rotating solid cylinder in the presence of a uniform magnetic field. Impact of the variations of undulations number (0 ≤ N ≤ 5), Ra (10^3^ ≤ Ra ≤ 10^6^), Ha (0 ≤ Ha ≤ 100), and angular rotational velocity (− 500 ≤ Ω ≤ 500) were presented. Isotherms distribution, streamlines and isentropic lines are displayed. The governing equations are verified by using the Galerkin Finite Element Method (GFEM). The Nusselt numbers are calculated and displayed graphically for several parametric studies. The computational calculations were carried out using Buongiorno's non-homogeneous model. To illustrate the studied problem, a thorough discussion of the findings was conducted. The results show the enhacement of the maximum value of the flow function and the heat transfer process by increasing the value of Rayleigh number. Furthermore the irreversibility is primarily governed by the heat transfer component and the increment of the waviness of the active surfaces or the cylinder rotational velocity or hartmann number will suppress the fluid motion and hinders the heat transfer process.

## Introduction

In recent years, enhancement of the heat transfer in industrial systems and engineering in general has become one of the most significant challenges of the twenty-first century^1–4^. Therefore, it is necessary to utilize modern technologies to overcome the problems and hindrances that limit the heat transfer process, different techniques can be employed to achieve this objective namely nanofluids, porous medium, magnetic fields, irregular walls, etc.

Nanofluid technologies are at the center of the interest of many investigators since their thermal conductivities are higher than conventional heat transfer fluids^4–7^. They present a very exciting advantage as they enable the enhancement of heat transfer rate of a thermal system without a single change to any of its components, and this can come about by simply suspending an iota of nanoparticles in a base fluid such as ethylene glycol, oil, and water. they have been used for the first time by Choi^8^ in 1995, since then many studies have been carried out to investigate their flow patterns and thermal behavior. Numerical research was conducted by Khanafer et al*.*^9^ to analyze the buoyancy-driven convection improvement of nanofluids in a 2D enclosure. Their outcomes explored that the nanoliquid thermal performance rate improves with an improvement in the solid concentration, the same model was adopted by Jou and Tzeng^10^ to examine the performance of nanoliquid heat transfer inside a rectangular cavity. Their results show that an increase in heat transfer rate is critical for nanofluids than pure fluids. Masoud et al*.*^11^ investigate natural convection in horizontal concentric annuli using different types of particles, they found that the high thermal conductivity of nanoparticles powerful improvement of convection phenomena. Otherwise, for intermediates Rayleigh number values, the low thermal conductivity of nanoparticles shows an adverse influence on heat transfer. Tiwari and Das^12^, studied numerically the convection parameters of nanoliquid in a two-sided lid-driven enclosure, They show that for both Richardson numbers and directions of moving walls modify heat transfer and fluid flow, it was also noted that solid volume fraction can change the fluid’s flow from free convection to forced convection regime. Sudarsana Reddy et al*.*^13^, carried out a numerical study on heat transfer and flow inside a square enclosure filled with SWCNTs—water nanofluid and using Finite difference scheme method, they found that the rates of heat transfer of the nanofluid increase as Rayleigh number values rises.

Moreover, thermal performance in a wavy cavity containing a rotating disk has been noticed by many researchers in recent decades. Combined convection flow across a heated wavy enclosure filled with Al_2_O_3_–H_2_O nanoliquid and containing a rotating cylinder were examined numerically by Alsabery et al*.*^14^, They found that in both directions of the rotating cylinder, the mean Nusselt number increased as Richardson's numbers increased. Hashim et al*.*^15^ were using Buongiorno’s model to investigate numerically the convection of alumina nanoliquid in a wavy enclosure with an inner conductive body, In their results, they found that the selection of the optimal number of the undulation leads to the convection improvement. Milani et al*.*^16^ in their study they studied the free convection mechanism in a nanofluids wavy cavity, they found that with an augmentation of the wavy amplitudes and wavy wavelengths, the strength of vortex decrease, thus Nusselt number decline.

Recently, a multitude of studies have been conducted on the influence of Lorentz forces on convective flow. Mokaddes et al*.*^17^ investigate the influence of Hartmann number on natural convection flow in a grooved cavity filled with nanoliquid. Dutta et al*.*^18^ carried out a CFD study on MHD natural convection heat transfer and entropy generation in a rhombic cavity. They found that the thermal convection decreases for increasing strength of Hartmann number. Sheikholeslami et al*.*^19^ modeled numerically on MHD flow through a permeable enclosure filled with alumina nanoliquid, in their analysis, they found that Lorentz forces impede the convection mode of heat transfer. Numerical research was conducted by Saha^20^. They show the effects of Hartmann number on the free convection of alumina nanoliquid in a differentially heated trapezoidal cavity with an inner circular obstacle, their results specified that the angle of externally applied field modifies significantly the streamline and isotherm profiles. Furthermore, the minimum value of Nu _loc_ slightly decreases with the progression of Hartmann number. Geridonmez and Oztop^21^ examined numerically the influence of cross partial magnetic fields on heat transfer and fluid flow in a cavity filled with nanofluid, they highlighted that the convective phenomena enhanced with the growth in the partial heater length while its decline with the strengthening of the Lorentz forces. Numerical analysis of nanofluid mixed convection inside a lid-driven grooved enclosure containing a circular solid and subjected by an external magnetic field and using a two-phase nanoliquid approach has been adopted by Alsabery et al*.*^22^, it has been noted that in case of natural convection is dominated, the including of solid particle can improve the thermal performance rate for the low value of the Reynolds number. Also, the addition of a magnetic affects the temperature distribution and nonliquid streamline.

Furthermore, the entropy generation due to irreversibility in thermal management results in energy loss. On other hand, it is necessary to perform a study on entropy generation to investigate the reasons and locations of irreversibility responsible for energy destruction. A Numerical study of entropy generation on mixed convection inside a corrugated wall lid-driven cavity filled with nanoliquid and subjected to an inclined magnetic force was carried out by Cho^23^. In their analysis, they show that irreversibility increases as solid concentration and wavy amplitude augment, and decrease as Hartmann number increases Sáchica et al*.*^24^ carried out a CFD study on mixed convection and irreversibility generated of alumina nanoliquid in a channel with two facing enclosures and separate heating in the presence of a Lorentz force, They observed that with a large number of volume fraction of nanoscale solid particles, the generation of entropy due to the magnetic effect decreases for all Ri and Ha numbers. Houshang et al*.*^25^ studied numerically natural convection and entropy generation into effect of a magnetic field in a trapezoidal cavity using Copper–water nanofluid. In their results, they found that the Bejan number is decreased as the nanoparticles are present, and increases with the increment of the magnetic field strength. Magnetohydrodynamic of mixed convection and entropy generation in a lid-driven enclosure containing rotating disk filled with nanoliquid and considering two-phase approach has been conducted by Barnoon et al*.*^26^ In their analysis, they observed that reducing Hartmann number, and increasing nanoparticle concentration, the magnitude of total entropy generation will improve. Mixed convection and entropy generation of Copper nanoparticles and pure water in a lid-driven enclosure have been analyzed by Khorasanizadeh et al*.*^27^ they revealed that the increasing and decreasing of entropy generations occur for both base fluid and solid particle, respectively, the introduction of nanoparticles in fluid incite the heat transfer rate more than improving entropy generation. For all values of the Ra number, the best selection of Re is important for the growth of thermal performance and entropy generation. Entropy generation and heat transfer analysis of magnetic hybrid nanofluid inside a square cavity with thermal radiation.

Generally, there are two basic approaches to simulate the nanoliquid flow and heat transfer mono-phase and two-phase and: the first model in which the nanoparticle and the basic liquid are in thermal equilibrium and have the same speed, it generally gives admissible results in a case of small volume fractions, But recently, researchers have turned to examine with a two-phase model because it gives more precise results than the mono-phase model due to the consideration of the Brownian motion effect and taken into account friction between the solid particles and fluid molecule, for example, analyze of mixed convection flow in lid-driven enclosure using two-phase mixture model was performed by Yu et al*.*^28^ considering the Brownian diffusion. Buongiorno^29^ has also implemented a two-phase mixture approach that accounts for Brownian motion and the thermoplastic effect of nanoliquid flow and formulated a two-component non-homogeneous equation for transport phenomena in nanofluids comprising the dominance of these two effects. This Buongiorno’s model was employed by Kefayati et al*.*^30^ to solve the mixed convection of nanofluid in an enclosure. A numerical investigation of Buongiorno’s nanofluid flow in a square cavity in the presence of a magnetic field was carried out by Sudarsana Reddy et al*.*^31^. They found that enhancing the thermophoresis and Brownian motion parameters improves the Nusselt number inside the cavity. Moreover, many other researchers have summarized reviews and studied the impact of different parameters on the flow and heat transfer characteristics in different cavities with different geometries including the impact of the magnetic field, porous media, rarefication effects, nanofluids and many others^32–37^.

As can be concluded from the litrature mentioned above, the mixed convection MHD flow of nanofluid were studied in different cases. Nevertheless, the literature on the mixed convection MHD nanofluid flow within a wavy cavity in the presence of an external magnetic force and a rotating cylinder is scarce. For this purpose, the authors are motivated to investigate numerically the combined effects of the of a centrally placed rotating cylinder and a magnetic field on the steady mixed convection two-phase flow of a (Al_2_O_3_–H_2_O) nanofluid, in this case the nanofluid flow is taken to be bounded between two vertical wavy walls and subjected to an imposed temperature gradient. The study will be performed using Galerkin Finite Element Method (GFEM). Effects of different parameters on flow and heat transfer will be presented and discussed thoroughly.

## Problem description

### Physical model

**Figure 1:**
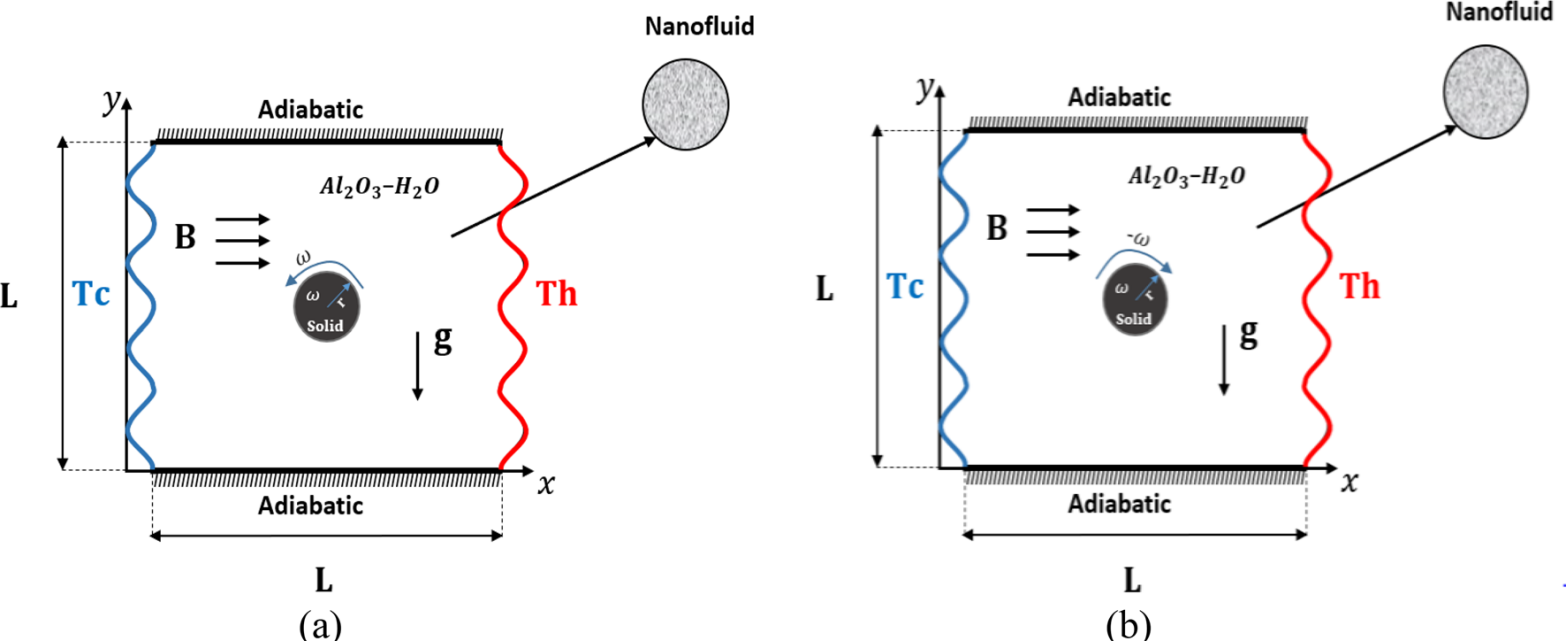
Physical model.

Figure 1 describes the problem of a 2D steady mixed convection in a wavy cavity in the presence of a central rotating cylinder with radius r, The inner moving rotating circular cylinder and both the horizontal walls are maintained as thermal insulation while the left and the right vertical walls are kept at the cold (Tc) and hot (Th) temperature respectively. The enclosure contains a Newtonian nanofluid having Al_2_O_3_ with water, The wavy walls equation executes Eq. (1)

1 $${y}_{left}=Acos\left(\frac{N\pi y}{L}\right),{y}_{Right}=-A[cos\left(\frac{N\pi y}{L}\right)-L],A=0.04\,and\,N=8$$

## The governing equations

Governing Eqs. (3), (4), (6),the continuity, energy equations and momentum for a laminar flow of a Newtonian fluid can be given as follows (see for details, Jou and Tzeng^10^):

2 $$\frac{\partial u}{{\partial x}} + \frac{\partial v}{{\partial y}} = 0,$$

3 $$\rho_{nf} \left( {u\frac{\partial u}{{\partial x}} + v\frac{\partial u}{{\partial y}}} \right) = - \frac{\partial p}{{\partial x}} + \mu_{nf} \left( {\frac{{\partial^{2} u}}{{\partial x^{2} }} + \frac{{\partial^{2} u}}{{\partial y^{2} }}} \right),$$

4 $$\rho_{nf} \left( {u\frac{\partial v}{{\partial x}} + v\frac{\partial v}{{\partial y}}} \right) = - \frac{\partial p}{{\partial y}} + \mu_{nf} \left( {\frac{{\partial^{2} v}}{{\partial x^{2} }} + \frac{{\partial^{2} v}}{{\partial y^{2} }}} \right) + g\beta_{nf} \rho_{nf} \left( {T - Tc} \right) - \sigma_{nf} B_{0}^{2} v$$

5 $$\left( {\rho Cp} \right)_{nf} \left( {u\frac{\partial T}{{\partial x}} + v\frac{\partial T}{{\partial y}}} \right) = k_{nf} \left( {\frac{{\partial^{2} T}}{{\partial x^{2} }} + \frac{{\partial^{2} T}}{{\partial y^{2} }}} \right) + Cp_{p} J_{p} \left( {\frac{\partial T}{{\partial x}} + \frac{\partial T}{{\partial y}}} \right)$$

6 $$u\frac{\partial \varphi }{{\partial x}} + v\frac{\partial \varphi }{{\partial y}} = D_{B} \left( {\frac{{\partial^{2} \varphi }}{{\partial x^{2} }} + v\frac{{\partial^{2} \varphi }}{{\partial y^{2} }}} \right) + D_{T} \left( {\frac{{\partial^{2} T}}{{\partial x^{2} }} + v\frac{{\partial^{2} T}}{{\partial y^{2} }}} \right),$$

Adopting Buongiorno’s model, the mass flux of nanoparticles (${\varvec{J}}_{p}$) is explained as the following^10^:

7 $${\varvec{J}}_{p} = {\varvec{J}}_{p,B} + {\varvec{J}}_{p,T} ,$$

8 $${\varvec{J}}_{p,B} = - \rho_{p} D_{B} \nabla \varphi { , }D_{B} = \left( {\frac{{k_{b} T}}{{3\pi \mu_{f} d_{p} }}} \right),$$

9 $${\varvec{J}}_{p,T} = - \rho_{p} D_{T} \nabla {\text{T, }}D_{T} = O.26\frac{{k_{f} }}{{2k_{f} + k_{p} }}\frac{{\mu_{f} }}{{\rho_{f} }}\frac{1}{T}\varphi {,}$$

The absolute velocity can be present as following:

10 $$\left|\mathrm{V}\right|=\left|\Omega \right|\mathrm{R}.$$

We have used in the current study the following thermophysical properties^28–30^:

**Heat capacitance:**

11 $${(\rho {C}_{p})}_{nf}={(\rho {C}_{p})}_{f}\left(1-\mathrm{\varphi }\right)+{(\rho {C}_{p})}_{p}\varphi ,$$

**Thermal diffusivity:**

12 $${\alpha }_{nf}=\frac{{k}_{nf}}{{\left(\rho {C}_{p}\right)}_{nf}},$$

**Density:**

13 $${\rho }_{nf}=\left(1-\varphi \right){\rho }_{f}+\varphi {\rho }_{p},$$

**Thermal expansion coefficient:**

14 $${(\rho \beta )}_{nf}={(\rho \beta )}_{f}\left(1-\varphi \right)+{(\rho \beta )}_{p}\varphi ,$$

**Dynamic viscosity ratio:**

15 $$\frac{{\mu }_{nf}}{{\mu }_{f}}=\frac{1}{{1-34.87\left(\frac{{d}_{p}}{{d}_{f}}\right)}^{-0.3}.{\phi }^{1.03}}$$

**Thermal conductivity ratio:**

16 $$\frac{{k}_{nf}}{{k}_{f}}=1+4.4{{Re}_{B}}^{0.4}{{P}_{r}}^{0.66}{(\frac{T}{{T}_{fr}})}^{10}{(\frac{{k}_{p}}{{k}_{f}})}^{0.03}{\phi }^{0.66}$$

${d}_{f}$ is the water’s molecular diameter which is given as Corcione^32^:

17 $${d}_{f}=(\frac{6M}{N\pi {\rho }_{f}})$$

The value of ${d}_{f}$ can be obtained as the following:

18 $${d}_{f}={\left(6\times \frac{0.01801528}{6.022}\times {10}^{23}\times \pi \times 998.26\right)}^\frac{1}{3}=3.85\times {10}^{-10},$$

The employed electrical conductivity ratio $\sigma_{nf} /\sigma_{f}$ can be described as Maxwell^32,33^:

19 $$\frac{{\sigma_{nf} }}{{\sigma_{f} }} = 1 + \frac{{3\left( {\frac{{\sigma_{p} }}{{\sigma_{f} }} - 1} \right)\varphi }}{{\left( {\frac{{\sigma_{p} }}{{\sigma_{f} }} + 2} \right) - \left( {\frac{{\sigma_{p} }}{{\sigma_{f} }} - 1} \right)\varphi }},$$

The dimensionless parameters are used:

20 $$\left. {\begin{array}{*{20}c} {X = \frac{x}{L}, Y = \frac{y}{L},{\text{U}} = \frac{{\text{u}}}{{{\text{U}}_{0} }} ,{\text{V}} = \frac{{\text{v}}}{{{\text{U}}_{0} }} P = \frac{{pL^{2} }}{{\rho_{nf} \vartheta_{f}^{2} }}, \varphi^{*} = \frac{\varphi }{\phi } ,} \\ {D_{B}^{*} = \frac{{D_{B} }}{{D_{B0} }},D_{T}^{*} = \frac{{D_{T} }}{{D_{T0} }},\delta = \frac{{T_{C} }}{{T_{h} - T_{C} }},\theta = \frac{{T - T_{C} }}{{T_{h} - T_{C} }},\Omega = \frac{{\omega L^{2} }}{{\alpha_{f} }}, R = \frac{r}{L}.} \\ \end{array} } \right\}$$

By using these dimensionless parameters, the equations become:

21 $$\frac{\partial U}{{\partial X}} + \frac{\partial V}{{\partial Y}} = 0,$$

$$U\frac{\partial U}{{\partial X}} + V\frac{\partial U}{{\partial Y}} = - \frac{\partial P}{{\partial X}} + \frac{{\rho_{f} }}{{\rho_{nf} }}\frac{{\mu_{nf} }}{{\mu_{f} }}\frac{1}{Re}\nabla^{2} U,$$

22 $$U\frac{\partial V}{{\partial X}} + V\frac{\partial V}{{\partial Y}} = - \frac{\partial P}{{\partial Y}} + \frac{{\rho_{f} }}{{\rho_{nf} }}\frac{{\mu_{nf} }}{{\mu_{f} }}\frac{1}{Re}\nabla^{2} V + \frac{{\left( {\rho \beta } \right)_{nf} }}{{\rho_{nf} \beta_{f} }}Ri\theta - \frac{{\rho_{f} }}{{\rho_{nf} }}\frac{{\sigma_{nf} }}{{\sigma_{f} }}\frac{{Ha^{2} }}{Re}V,$$

23 $$\begin{gathered} U\frac{\partial \theta }{{\partial X}} + V\frac{\partial \theta }{{\partial Y}} = \frac{{\left( {\rho Cp} \right)_{f} }}{{\left( {\rho Cp} \right)_{nf} }}\frac{{k_{nf} }}{{k_{f} }}\frac{1}{{{\text{Re}} \cdot \Pr }}\left( {\frac{{\partial^{2} \theta }}{{\partial X^{2} }} + \frac{{\partial^{2} \theta }}{{\partial Y^{2} }}} \right) + \frac{{\left( {\rho Cp} \right)_{f} }}{{\left( {\rho Cp} \right)_{nf} }}\frac{{D_{B}^{*} }}{{{\text{Re}} \cdot \Pr \cdot {\text{Le}}}}\left( {\frac{{\partial \varphi^{*} }}{\partial X}\frac{\partial \theta }{{\partial X}} + \frac{{\partial \varphi^{*} }}{\partial Y}\frac{\partial \theta }{{\partial X}}} \right) \hfill \\ + \frac{{\left( {\rho Cp} \right)_{f} }}{{\left( {\rho Cp} \right)_{nf} }}\frac{{D_{T}^{*} }}{{{\text{Re}} \cdot \Pr \cdot {\text{Le}} \cdot N_{BT} }}\frac{1}{1 + \delta \theta }\left( {\left( {\frac{\partial \theta }{{\partial X}}} \right)^{2} + \left( {\frac{\partial \theta }{{\partial Y}}} \right)^{2} } \right)^{2} , \hfill \\ \end{gathered}$$

24 $$U\frac{{\partial \varphi^{*} }}{\partial X} + V\frac{{\partial \varphi^{*} }}{\partial Y} = \frac{{D_{B}^{*} }}{{{\text{Re}} \cdot Sc}}\left( {\frac{{\partial^{2} \varphi^{*} }}{{\partial X^{2} }} + \frac{{\partial^{2} \varphi^{*} }}{{\partial Y^{2} }}} \right) + \frac{{D_{T}^{*} }}{{{\text{Re}} \cdot Sc \cdot N_{BT} }}\frac{1}{1 + \delta \theta }\left( {\frac{{\partial^{2} \theta }}{{\partial X^{2} }} + \frac{{\partial^{2} \theta }}{{\partial Y^{2} }}} \right),$$

### Description of the embedded control physical parameters

Where $D_{T0} = 0.26 \frac{{k_{f} }}{{2k_{f} + k_{p} }}\frac{{\mu_{f} }}{{\rho_{f} \theta }}\phi$ and $D_{B0} = \frac{{k_{b} T_{C} }}{{3\pi \mu_{f} d_{p} }}$ are thermophoretic diffusion and Brownian diffusion coefficient, $S_{C} = \frac{{v_{f} }}{{D_{B0} }}$ is Schmidt number, $N_{BT} = \frac{{\phi D_{B0} T_{C} }}{{D_{T0} \left( {T_{h} - T_{C} } \right)}}$ is the ratio of diffusivity parameter between Brownian diffusivity and thermophoretic diffusivity, $L_{e} = \frac{{k_{f} }}{{\left( {\rho C_{p} } \right)_{f} \phi D_{B0} }}$ is Lewis number, $Ri = \frac{Gr}{{{\text{Re}}^{2} }}$ is Richardson number, $Gr = \frac{{g\beta_{f} L^{3} \left( {T_{h} - T_{C} } \right)}}{{\upsilon_{f}^{2} }}$ is Grashof number and ${\text{Re}} = \frac{{U_{0} L}}{{\nu_{f} }}$ is Reynolds number. $Pr = \frac{{\upsilon_{f} }}{{\alpha_{f} }}$ is Prandtl number and $Ha^{2} = B_{0}^{2} L^{2} \frac{{\sigma_{f} }}{{\mu_{f} }}$ is Hartmann number.

The dimensionless boundary conditions of equations are given as flowing:

On the rotating solid cylinder surface:

$$U=-\Omega \left(Y-{Y}_{0}\right),V=\Omega \left(X-{X}_{0}\right),$$

25 $$\frac{{\partial \varphi^{*} }}{\partial n} = 0,\frac{\partial \theta }{{\partial n}} = 0$$

On the right hot wall of the cavity:

26 $$U = V = 0,\frac{{\partial \varphi^{*} }}{\partial n} = - \frac{{D_{T}^{*} }}{{D_{B}^{*} }}.\frac{1}{{N_{BT} }}.\frac{1}{1 + \delta \theta }\frac{\partial \theta }{{\partial n}}, \theta = 1$$

On the left cold wall of the cavity:

27 $$U = V = 0,\frac{{\partial \varphi^{*} }}{\partial n} = - \frac{{D_{T}^{*} }}{{D_{B}^{*} }}.\frac{1}{{N_{BT} }}.\frac{1}{1 + \delta \theta }\frac{\partial \theta }{{\partial n}}, \theta = 0$$

On the adiabatic horizontal walls:

28 $$U = V = 0, \frac{{\partial \varphi^{*} }}{\partial n} = 0,\frac{\partial \theta }{{\partial n}} = 0$$

Local Nusselt number does estimated toward the heated right wall vertical surface of the cavity as:

29 $$Nu_{nf} = - \frac{{k_{nf} }}{{k_{f} }}\left( {\frac{\partial \theta }{{\partial X}}} \right)_{X = 0} .$$

The mean Nusselt number $\overline{{N }_{u}}$ is obtained by integrating the local Nusselt number:

30 $$\overline{{Nu_{nf} }} = \int\limits_{0}^{1} {Nu_{nf} {\text{dY}}} .$$

The entropy generation is given by^22^:

31 $$S_{gen} = \frac{{k_{nf} }}{{T_{0}^{2} }}\left[ {\left( {\frac{\partial T}{{\partial x}}} \right)^{2} + \left( {\frac{\partial T}{{\partial y}}} \right)^{2} } \right] + \frac{{\mu_{nf} }}{{T_{0} }}\left[ {2\left( {\left( {\frac{\partial u}{{\partial x}}} \right)^{2} + 2\left( {\frac{\partial v}{{\partial Y}}} \right)^{2} } \right) + \left( {\frac{\partial u}{{\partial y}} + \frac{\partial v}{{\partial x}}} \right)^{2} + \frac{{\sigma_{nf} B^{2} V^{2} }}{{T_{0} }}} \right].$$

Using dimensionless form, entropy generation can be assumed as following:

32 $$S_{GEN} = \frac{{k_{nf} }}{{k_{f} }}\left[ {\left( {\frac{\partial \theta }{{\partial X}}} \right)^{2} + \left( {\frac{\partial \theta }{{\partial Y}}} \right)^{2} } \right] + \frac{{\mu_{nf} }}{{\mu_{f} }}N_{\mu } \left[ {2\left( {\left( {\frac{\partial U}{{\partial X}}} \right)^{2} + 2\left( {\frac{\partial V}{{\partial Y}}} \right)^{2} } \right) + \left( {\frac{\partial U}{{\partial Y}} + \frac{\partial V}{{\partial X}}} \right)^{2} + N_{\mu } \frac{{\sigma_{nf} }}{{\sigma_{f} }}Ha^{2} V^{2} } \right].$$

where, ${N}_{\mu }=\frac{{\mu }_{f}{T}_{0}}{{k}_{f}}{\left(\frac{{\alpha }_{n}}{L\left(\Delta T\right)}\right)}^{2}$ presented as the ratio irreversibility of distribution and the terms of Eq describe discretely in the following form:

33 $$S_{GEN} = S_{HT} + S_{FF} + S_{MF} .$$

where ${S}_{HT}$, ${S}_{FF}$, ${S}_{MF}$ describe the entropy production, of the heat transfer irreversibility (HTI), nanoliquid friction irreversibility (NFI) and entropy generation due to magnetic effect (SMF), respectively.

34 $$S_{HT} = \frac{{k_{nf} }}{{k_{f} }}\left[ {\left( {\frac{\partial \theta }{{\partial X}}} \right)^{2} + \left( {\frac{\partial \theta }{{\partial Y}}} \right)^{2} } \right],$$

35 $$S_{FF} = \frac{{\mu_{nf} }}{{\mu_{f} }}N_{\mu } \left\{ {2\left[ {\left( {\frac{\partial U}{{\partial X}}} \right)^{2} + 2\left( {\frac{\partial V}{{\partial Y}}} \right)^{2} } \right] + \left( {\frac{{\partial^{2} U}}{{\partial Y^{2} }} + \frac{{\partial^{2} V}}{{\partial X^{2} }}} \right)^{2} } \right\},$$

36 $$S_{MF} = N_{\mu } \frac{{\sigma_{nf} }}{{\sigma_{f} }}Ha^{2} V^{2} .$$

The dimensionless average generation entropy *S*avg can be calculated by integrating Eq. (36) as:

37 $$S_{avg} = \frac{1}{V}\int {S_{GEN} dV} = S_{HT.avg} + S_{FF.avg} + S_{MF,avg}$$

Here *V* denotes total volume of the nanoliquid, Where $S_{HT.avg} ,\,S_{FF.avg}$ and $\,S_{MF,avg}$ present the average entropy generation due to heat transfer irreversibility, nanofluid friction irreversibility and magnetic effect, respectively.

In addition, Bejan number determines which is the dominant, heat transfer or nanoliquid friction irreversibility^22^:

38 $$B_{e} = \frac{{S_{HT} }}{{S_{GEN} }}.$$

The thermo physical properties of base fluid water and Aluminium oxide–water (Al_2_O_3_–H_2_O) is defined in Table 1.

**Table 1:** Thermo-physical characteristics of base fluid (H_2_O) and nanoparticles (Al_2_O_3_)^19^.

Physical characteristics	Al_2_O_3_	H_2_O
C_p_ (J/kg k)	765	4179
$\rho$ (kg/m^3^)	3970	997.1
K (W/m k)	25	0.613
β × 10^–5^ (K^−1^)	0.85	21
Σ (S/m)	${10}^{-10}$	0.05

## Validation and grid independency

The governing dimensionless Eqs. (21), (22), (23) and (24) together with the boundary conditions are computed using Galerkin weighted residual finite element method^38,39^. Briefly, the fist step in this method is to discretize the solution domain into a limited number of meshes, which are clusters of non-uniform triangular elements. To assure the mesh independence of the numerical solution, different meshes were taking to determine the maximum value of the flow circulation (${\Psi }_{max}$), the mean Nusselt number ($\overline{{N }_{u}}$) at Ra = 10^4^, Ha = 25, Ω = 0 and N = 5.

**Figure 2:**
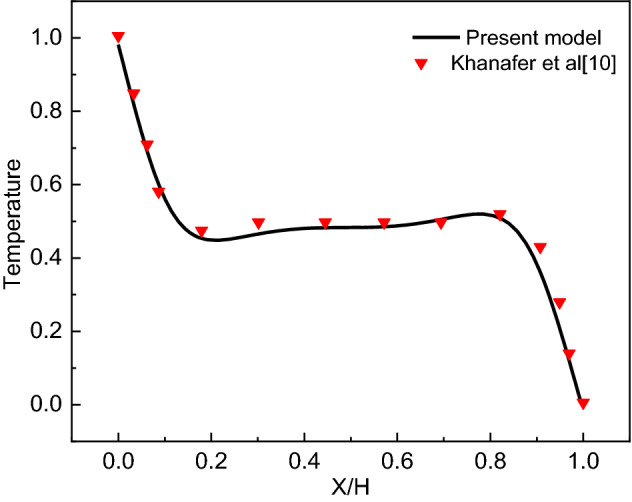
Dimensionless temperature distribution for (Ra = 10^5^, Pr = 0.7).

Table 2 displays that the difference between the solutions on grids grid 6 and grid 7 is inconsequential. Thus, we adopted grid 6 in all numerical simulations presented in this work. The present model has been validated against Khanafer et al*.*^9^ results (see Fig. 2).

**Table 2:** Grid independent test for ${\Psi }_{max}$ and $\overline{{N }_{u}}$ at Ra = 10^4^, Ha = 25, Ω = 0 and N = 5.

Size	Elements	${\Psi }_{max}$	$\overline{{N }_{u}}$
G1	2074	3.4474	2.0037
G2	3252	3.5144	1.9894
G3	3962	3.5220	1.9879
G4	5730	3.5440	1.9822
G5	8924	3.5472	1.9813
G6	22,734	3.5524	1.9717
G7	31,858	3.5549	1.9716

## Results and discussion

The previously described setup sets the stage to a more detailed investigation into the affecting factors and their role on the outputs. Outputs include fluid motion studied through the observation of the resulting streamlines, the thermal distribution studied through the isotherm contours, and finally entropy distribution through studying the isentropic contours. The affecting factors include the geometric factor characterized by the undulation number, the fluid flow characterized by the Rayleigh number, the magnetic intensity to which the nanofluid is subjected characterized by the Hartmann number, and the rotation of the cylinder characterized by its rotational speed. The numerical observations are to be conducted for Rayleigh numbers range of values (10^3^ ≤ Ra ≤ 10^6^), the cylinder angular rotational speed in the range (− 500 ≤ Ω ≤ 500), and the Hartmann number in the range of (0 to100).

**Figure 3:**
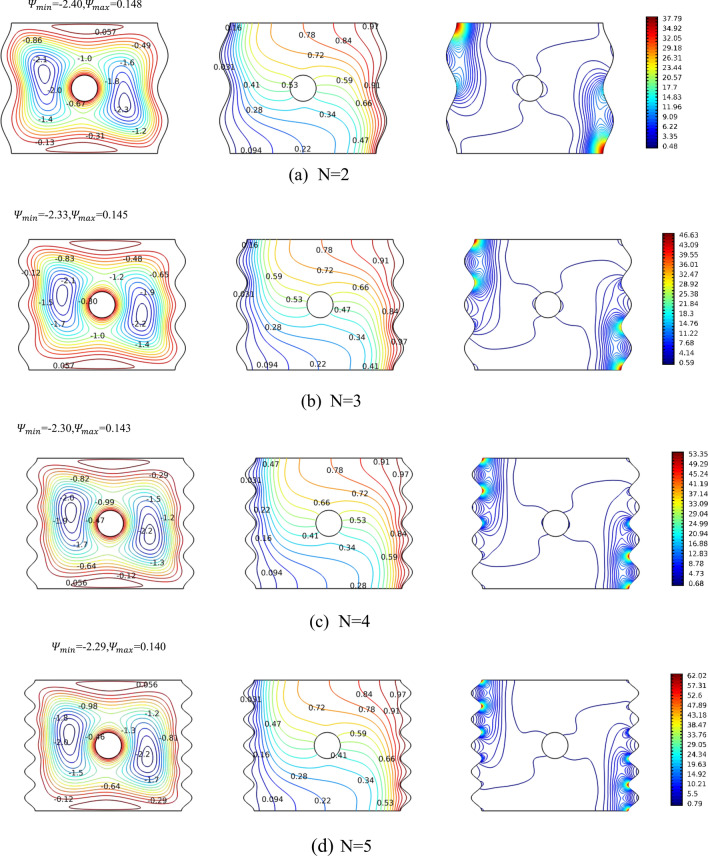
Streamlines, temperature contours and isentropic variation for various undulations (N) for case Ra = 10^4^, Ha = 0, Ω = 0. The panels represent the levels of the contours.

In the first investigation, studying magneto-free convection is accomplished into a nanofluid cavity along with a conductive circular non-rotating impediment for various cavity’s cross section’s wavy surface. Mainly, the effect of the waviness is observed by changing the undulation number from 2 to 5, at Ra = 10,000 (Fig. 3). The first column, which represents the stream function contours, shows that in all cases there exists two sets of concentric contours on the right and left of the cylinder. Though the centers are not aligned horizontally with the symmetry line tilted from vertical, where the tilting is caused by the fact that the right side of the cavity is hot whereas the left side is cold which impacts the fluid motion. Interesting to observe that at N = 2, the symmetry line is tilted at 19° from vertical line and reduced for N = 3 to 13°, where finally for N = 4 and N = 5 the tilt is maintained at an intermediate value of 16°–17°, which means with stationary cylinder, increasing the undulation number after 4 is not impacting the center of the stream lines contours and motion. The values of the stream functions for all cases are very close to each other giving the hint that the undulation number does not significantly impact the fluid flow under such circumstances (Ra = 10,000, and zero rotation and magnetic fields). As the flow is minimally impacted, the isotherms in the second column of Fig. 3 shows also insensitivity in reference to the undulation number. It is mainly described as being with high density near the heat source and sink (left and right sides) taking its wavy shape, and scarcely distributed in the bulk of the cavity. The scarcity is reduced minimally for larger N values, meaning that the average Nusselt number will decrease with N as will be seen later, with emphasis again on minimal change. Finally for the entropy (isentropic) contours, it can be seen there is a symmetry diagonally established. The density of the isentropic lines increases at the sides and decreases in the middle. The number of “hot spots” (in red) representing the largest values of the isentropic contours is a function of the undulation number. When N increases, more high value concentrations appear towards horizontal center line.

**Figure 4:**
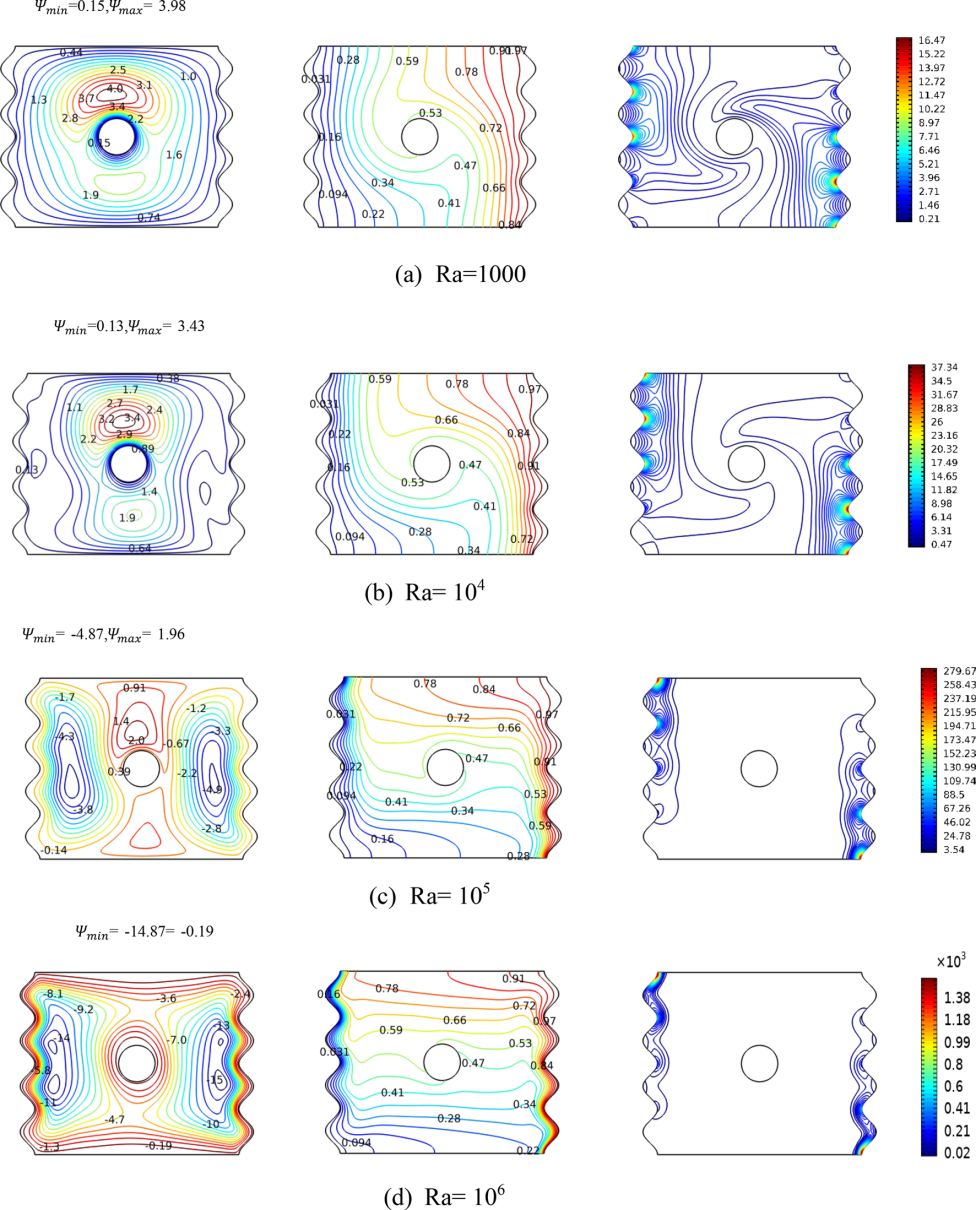
Streamlines, isotherms and isentropic variation by Ra for case, Ha = 25, Ω = 250. The panels represent the levels of the contours.

In the second investigation, the geometry of the cross section is fixed at N = 4, subjected to relatively small magnetic field with Ha = 25 and under the effect of a rotating cylinder with rotational speed of 250 whereas the flow is varied by increasing the Raleigh number from 1000 to 1,000,000 wich is a good indication of the increase in the bounancy forces (see Fig. 4). As Ra impacts the flow significantly by moving the heat transfer characteristics from conduction mode of heat transfer to convective one, the first column in Fig. 3 shows the tremendous evolution of the streamlines with Ra. At low Ra value which represents conduction mode of heat transfer, the highest stream function value is upward of the cylinder indicating the dominancy of the integration of the impact of the rotation of the cylinder with the magnetic field effect. Increasing Ra will tend to move the extreme stream function values towards the left and the right sides of the enclosure creating dominant vortices there, observing for Ra = 10^6^ where the convection mode of heat transfer is dominant and just at the end boundary of laminar flow, the effect of the rotating cylinder and the magnetic field is local. All of this, especially the rise of vortices near heat source and sink predicts enhanced heat transfer. Therefore, one can observe the isotherms (middle column in Fig. 4) evenly distributed over the cavity for low Ra, whereas it takes the shape of the most affecting heat transfer for high Ra, where the isotherms lines are highly dense near the hot and cold sides and scarce towards the middle of the cavity, which increases the thermal gradient; the driving force of the heat transfer.

The isentropic contours iterate identical tale. As mentioned previously, the generated irreversibility is comprised of 3 components: heat transfer, nanofluid friction, and magnetic effect. At low Ra values, the three components are comparable and hence the isentropic lines span the entire cavity. However, with increased Ra. the heat transfer component becomes the dominant and thus the isentropic contours are converging to the active (thermally) sides and disappearing from the middle.

**Figure 5:**
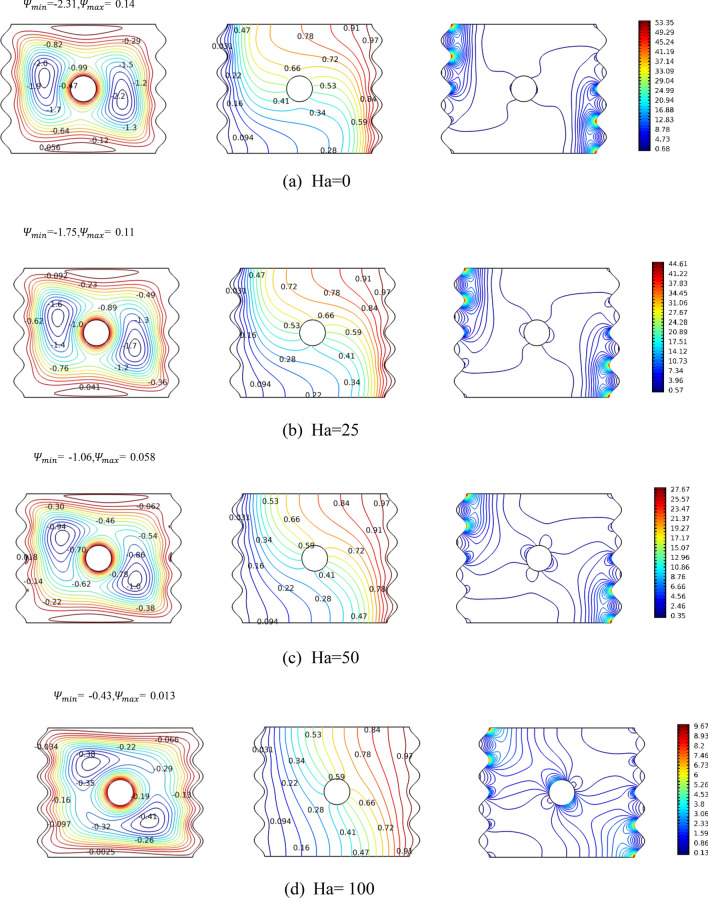
Streamlines, isotherms and isentropic variation by Hartman number Ha for case, φ = 0.04, Ra = ${10}^{4}$, Ω = 0. The panels represent the levels of the contours.

Figure 5 demonstrates the investigation of the thermofluidic operations within the cavity when the magnetic field intensity is varied for the case of Ra = 10^4^, N = 4, and no rotation for the cylinder. The Ha variations take place as follows: 0, 25, 50, and 100. The nanofluid is impacted by the magnetic field where the creation of Lorentz force hinders the fluid motion. That can be clearly seen from the first column where the extreme value for the streamline function decreases with Ha, reaching as low as 81% reduction when comparing Ha = 100 case with no magnetic field case. Nonetheless, it should be pointed out that due to the cylinder being stationary, the location of the vortices is minimally changed. Isotherms wise (second column of Fig. 5), the density of the lines are expectedly large at the thermally active sides where they become less dense towards center of cavity. The difference between the first three cases (Ha = 25, and 50) is very small predicting the difference in their effective heat transfer is very small. However, for the case of Ha = 100, the magnetic field is hindering the fluid motion significantly and thus it can be seen the isotherms contours almost evenly distributed as if it is heat transfer by conduction only. Finally, the isentropic contours show the significance of hindering the fluid motion where the more Ha is, the larger the area they cover within the cavity’s cross section. The components of the entropy generation and irreversibility by the nanofluid friction and magnetic field are assuming significant portions of the total irreversibility. In general, as the magnetic field increases, the flow is suppressed and the heat transfer is reduced.

**Figure 6:**
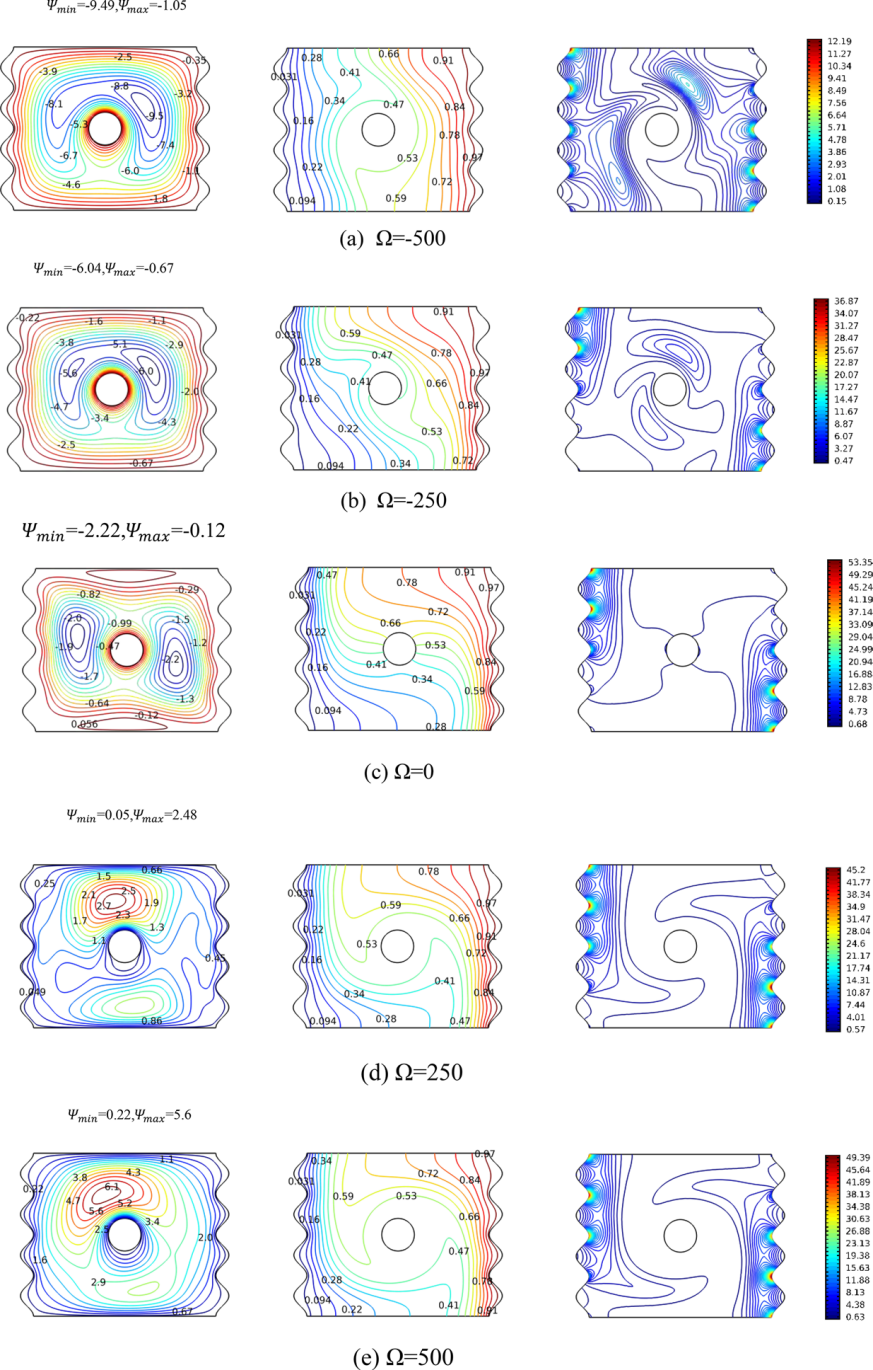
Streamlines, isotherms and isentropic variation by angular rotational velocity Ω for Ra = 10^4^, Ha = 0, N = 4. The panels represent the levels of the contours.

Figure 6 investigates the role of the cylinder’s rotation in the thermofluidic operations at Ra = 10^4^, N = 4, and zero magnetic field. The investigation includes both the rotational speed magnitude and direction. For the first column, the cylinder’s rotation effect on the stream lines is evident. While the stationary cylinder shows slightly tilted left and right vortices (as discussed upwards in reference to Fig. 3), once the cylinder rotates these vortices centers move and the streamlines shapes are impacted. For example, the first two represent clockwise rotations, meaning more and more focus on the right vortex and pushing it upward. The natural convection-based nanofluid motion from the bottom right hot surface to the upper part of the left cold one (against gravity) is rotated back to the cold surface by the clockwise rotation but this time to the upper part. That creates that continuously becoming stronger (with more rotational speed) vortex towards the right top part of the cavity. On the contrary, rotating the cylinder counter clockwise (as in + 250 and + 500) moves the vortices to almost become upward and downward of the cylinder with the stream function values being much higher for the upper one. Mainly, the fluid near the bottom of the hot surface is trying to travel by natural convection towards the top of the cold surface; however, it is countered by the hot fluid from the top of the right hot surface that is pushed by the rotation of the cylinder creating the easily observed upward strong vortex. The story of the isotherms is less divergent. That is the isotherm contours differ amongst different rotational speed but not in an extreme manner. The negative clockwise rotation shows less densities of isotherms near the active surfaces meaning their heat transfer is not effective. On the contrary, the positive counter clockwise rotations exhibit isotherms dense at the active surfaces and slightly spars in the bulk of the cavity, similar to the stationary case, so their effective heat transfer will be higher than clock wise one. In all matters, the isotherms indicate comparable heat transfer rates amongst all rotations as will be shown later in Fig. 6 for the case of Ra = 10^4^. The isentropic lines once again confirm the story of the isotherms where the cases of stationary and positive rotations drawings, the contours concentrations are very similar. However, rotating the cylinder in a clockwise manner creates new contours near the cylinder becoming more visible for the high velocity’s clockwise rotation. That is the entropy generation in the stationary and counter clock wise rotations are governed by the heat transfer irreversibility, whereas the nanofluid friction is exhibiting more contribution in the clockwise rotation, evident by the contours created away from the active sides.

**Figure 7:**
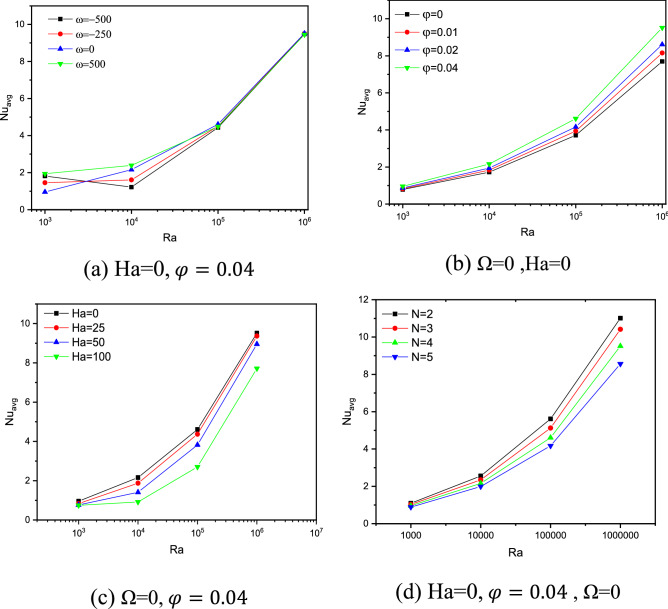
Impact of Ra on ${Nu}_{avg}$ with varying Ω (**a**), $\varphi$ (**b**) and Ha (**c**) at N = 4, N (**d**).

The isotherms give insights to the ongoing heat transfer, but sometimes one would like to compare effective heat transfers with a single value. That value is the average Nusselt number. Figure 7 provides parametric investigations of the relation between the Nu_avg_ with Ra for 4 different parameters: cylinder rotational speed (Ω), the nanoparticles volume fraction (ϕ), the magnetic intensity characterized by the Hartmann number (Ha), and the cavities cross sectional geometry characterized by the undulation number (N).

**Figure 8:**
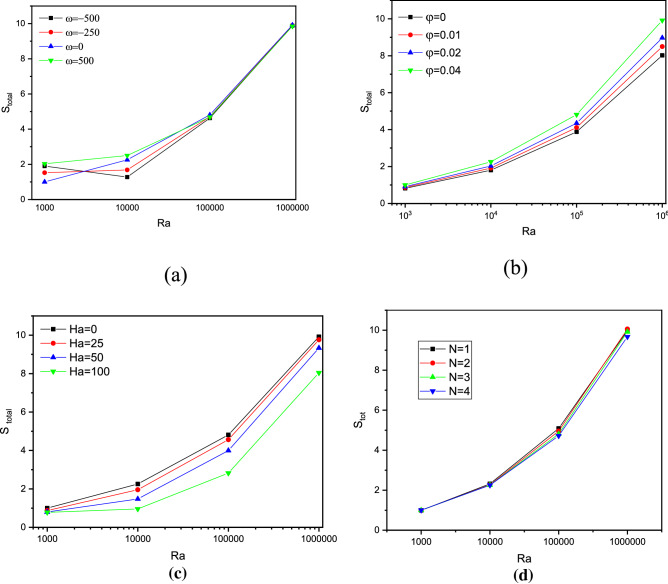
Impact of Ra on ${S}_{Total}$ function with varying Ω (**a**), $\varphi$ (**b**) and Ha (**c**) at N = 4.

The plain observation is that with increasing Ra, more fluid motion is occurring enabling the effective part of the heat transfer, which the convection part, to become more impacting and achieving higher Nu_avg_. Focusing on Fig. 7a, the high Ra values represent a very high fluid motion such that it becomes blind to the cylinder’s rotational speeds (magnitude and direction). Though for Ra = 10^4^, the case to which the isotherms were analyzed in Fig. 6, we have predicted that the heat transfer is comparable with slight edge to the stationary and counter clockwise rotations. For the small Ra value of 1000, the motion is displaying small motions that the cylinder rotations and, in any direction, actually increases Nu_avg_.

In Fig. 7b, the nanoparticles concentration shows enhancement of the Nu_avg_, especially for higher Ra values. For the case of Ra = 10^6^, the enhancement from ϕ = 0 to 0.04 is as high as 24.7%, whereas for Ra = 1000, the enhancement is barely seen. With high fluid motion, the actively moving nanoparticles enhance the heat transfer process and their volume fraction means more volume and more heat carrying vibrant masses. It is though expected that this behavior to increase up to a limit where the volume of the nanoparticles becomes a hindering factor rather than a catalyst.

In Fig. 7c, the previously discussed case of Fig. 4 regarding the influence of the magnetic field is shown. The magnetic field impacts the nanofluid motion slowing it down and thus its increase always has a negative effect on the effective heat transfer characterized by the Nu_avg_. The former statement is valid for all ranges of Ra.

Finally in Fig. 7d, the impact of the undulation number on the Nu_avg_ is presented. Observing the second set of the bins (Ra = 10^4^) which matches the detailed case studied and discussed in Fig. 2, the change with N is small with a decrease of 0.6 in Nu_avg_ when is N is increased from 2 to 5. However, the undulation number at the case of the last set of bins (Ra = 10^6^) reduces Nu_avg_ by 2.35 when is N is increased from 2 to 5. Though it should be pointed out that the percentage of reduction is the same at 22–23% for both Ra values.
